# Supplementary material for: The natural history of the emergence of sexually transmissible shigellosis
Source: Microb Genom. 2026 Jan 21;12(1):001607. doi: 10.1099/mgen.0.001607 (PMC12824643; doi:10.1099/mgen.0.001607)
Supplement: Uncited Supplementary Material 1. [file mgen-12-01607-s001.pdf]

Supplementary Information

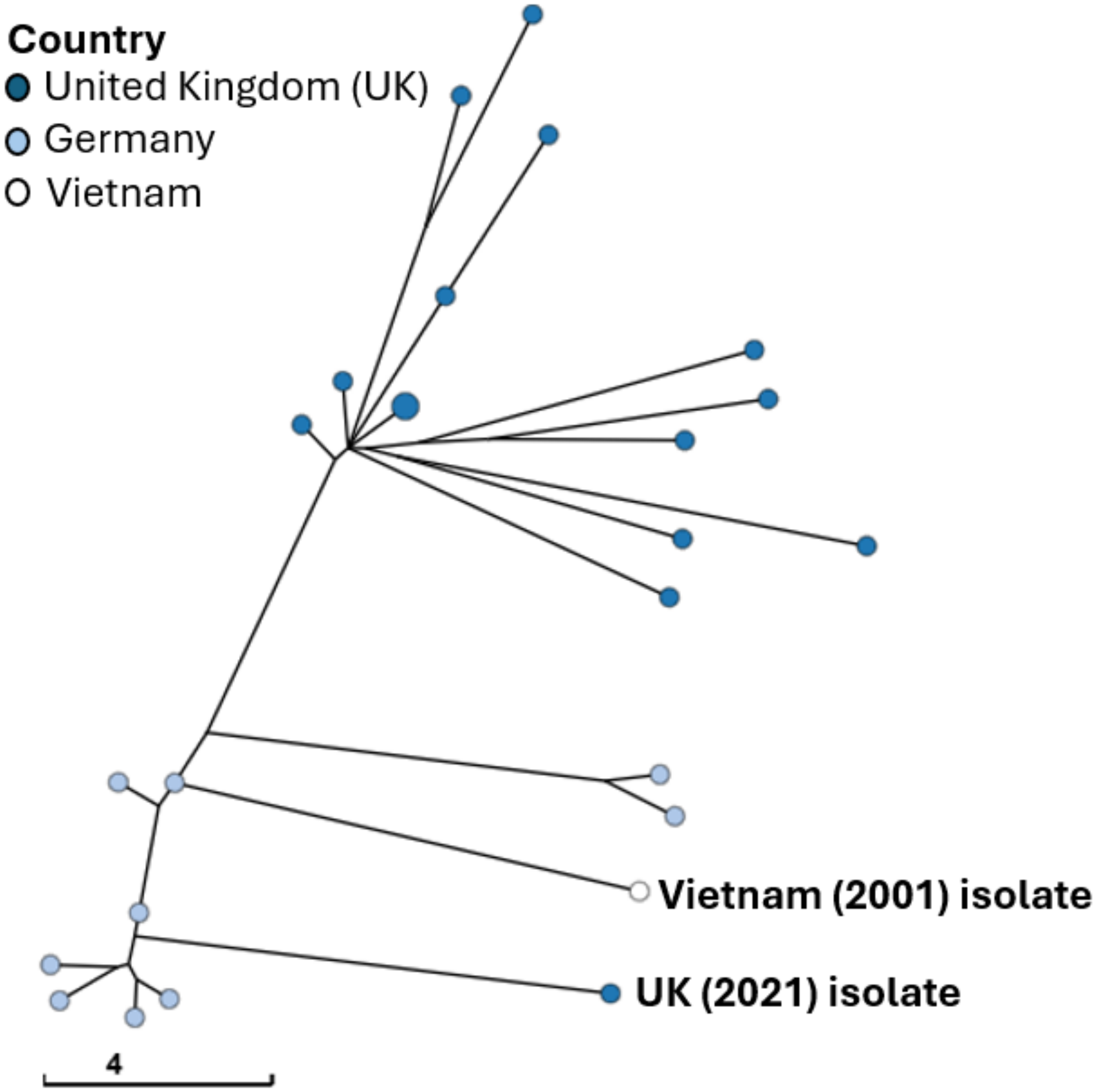

Supplementary Figure 1. Enterobase v1.2.0 cgMLST + HierCC Ninja NJ tree of the London and Berlin outbreak isolates, alongside two further isolates identified in NCBI Pathogen Detection to be genetically similar. The scale bar represents number of cgMLST allelic differences.

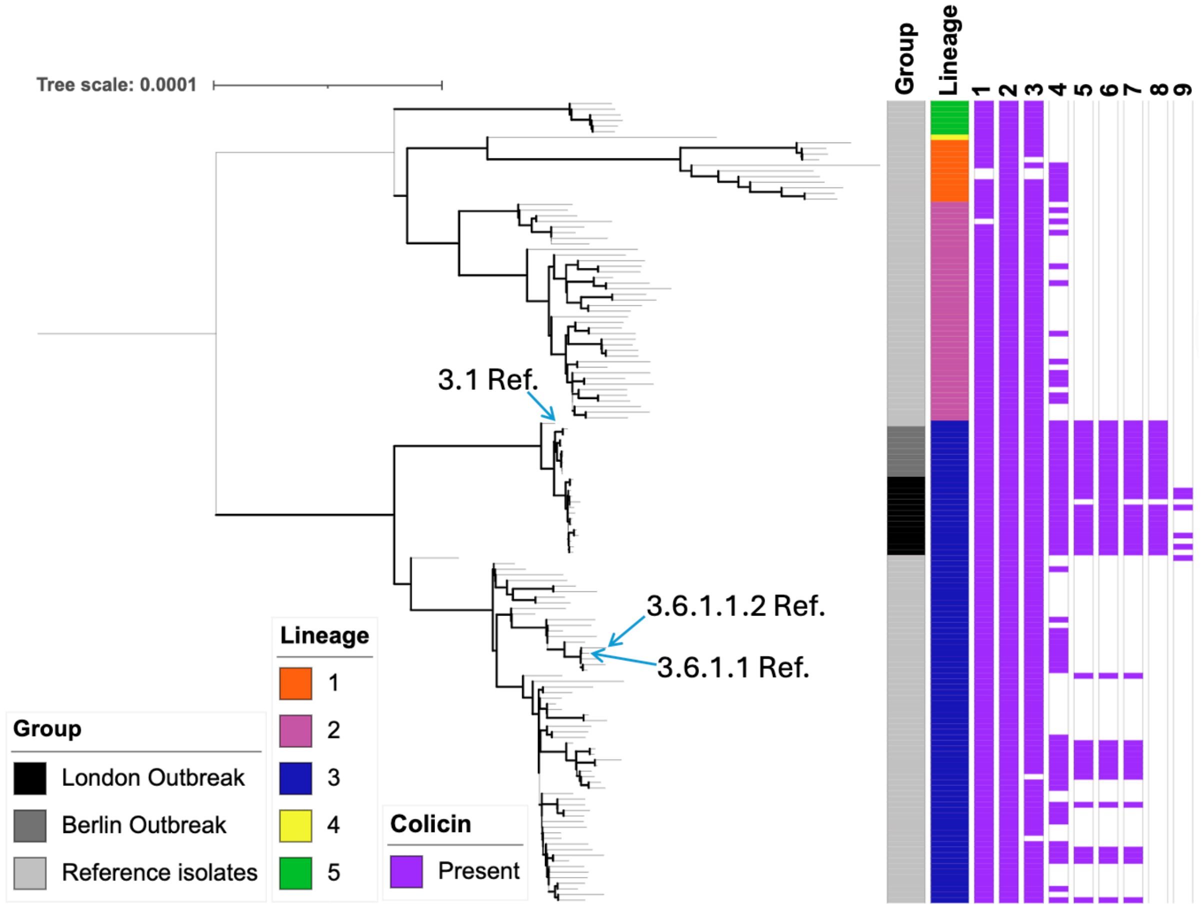

Supplementary Figure 2. The London and Berlin outbreak isolates have a conserved colicin gene profile. The phylogenetic tree in Figure 4 with metadata tracks showing: group, lineage, and presence or absence of colicin genes which changed in proportions among groups, coloured according to the inlaid keys. Colicin genes have been represented as lane numbers, corresponding to the following: (1 = Colicin, ENA | QPR02973), (2 = Colicin Import Protein, ENA | CDU39502), (3 = Colicin-I-Receptor, ENA | EGI15668), (4 = Colicin Lysis Protein Precursor, ENA | AAN28375), (5 = Colicin T7-1, ENA | AAK67298), (6 = Colicin T7-3, ENA | CAC41014), (7 = Colicin Type 7, ENA | AAK67300), (8 = Colicin, ENA | EFY5874042), (9 = Colicin, ENA | QLM32732).

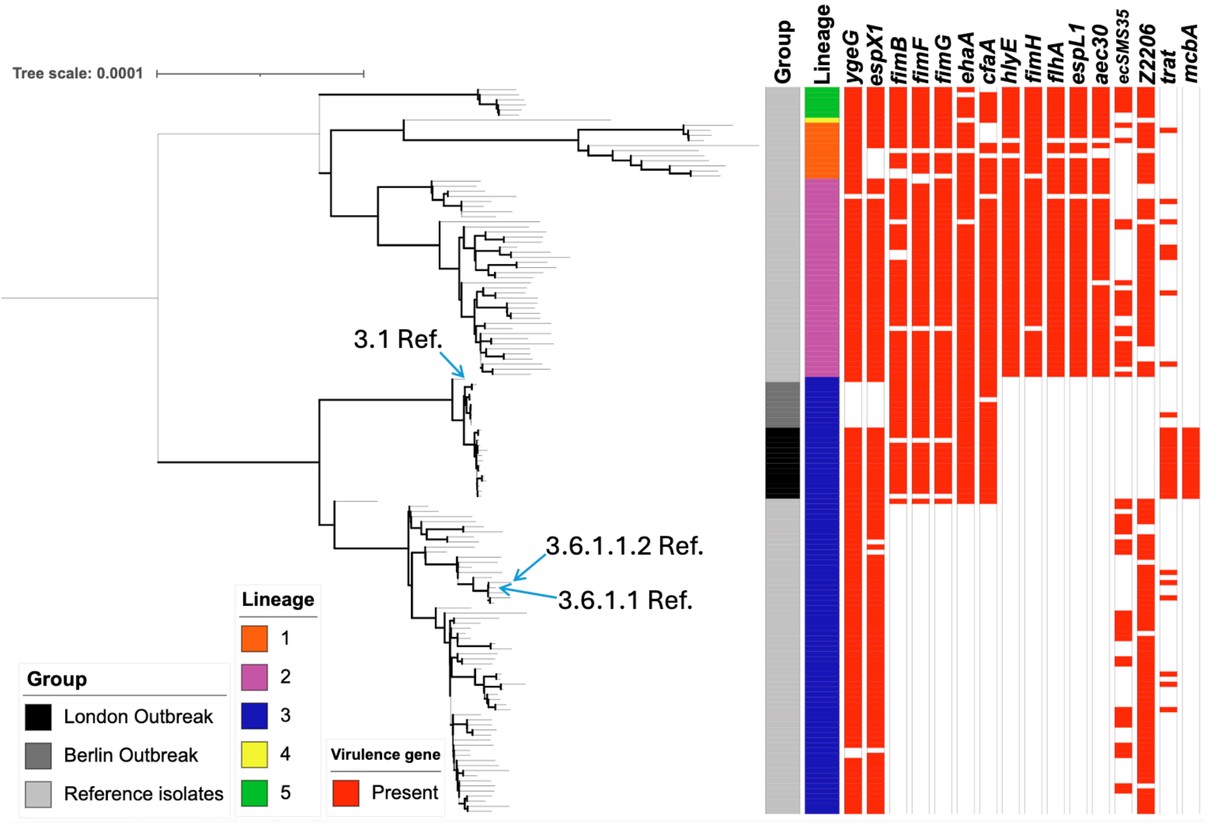

Supplementary Figure 3. Virulence gene loss and acquisition among the London and Berlin outbreak isolates. The tree from Figure 4 is shown with metadata tracks showing, group, Lineage, and the presence or absence of selected virulence genes which change in proportion among groups of interest, coloured according to the inlaid keys.

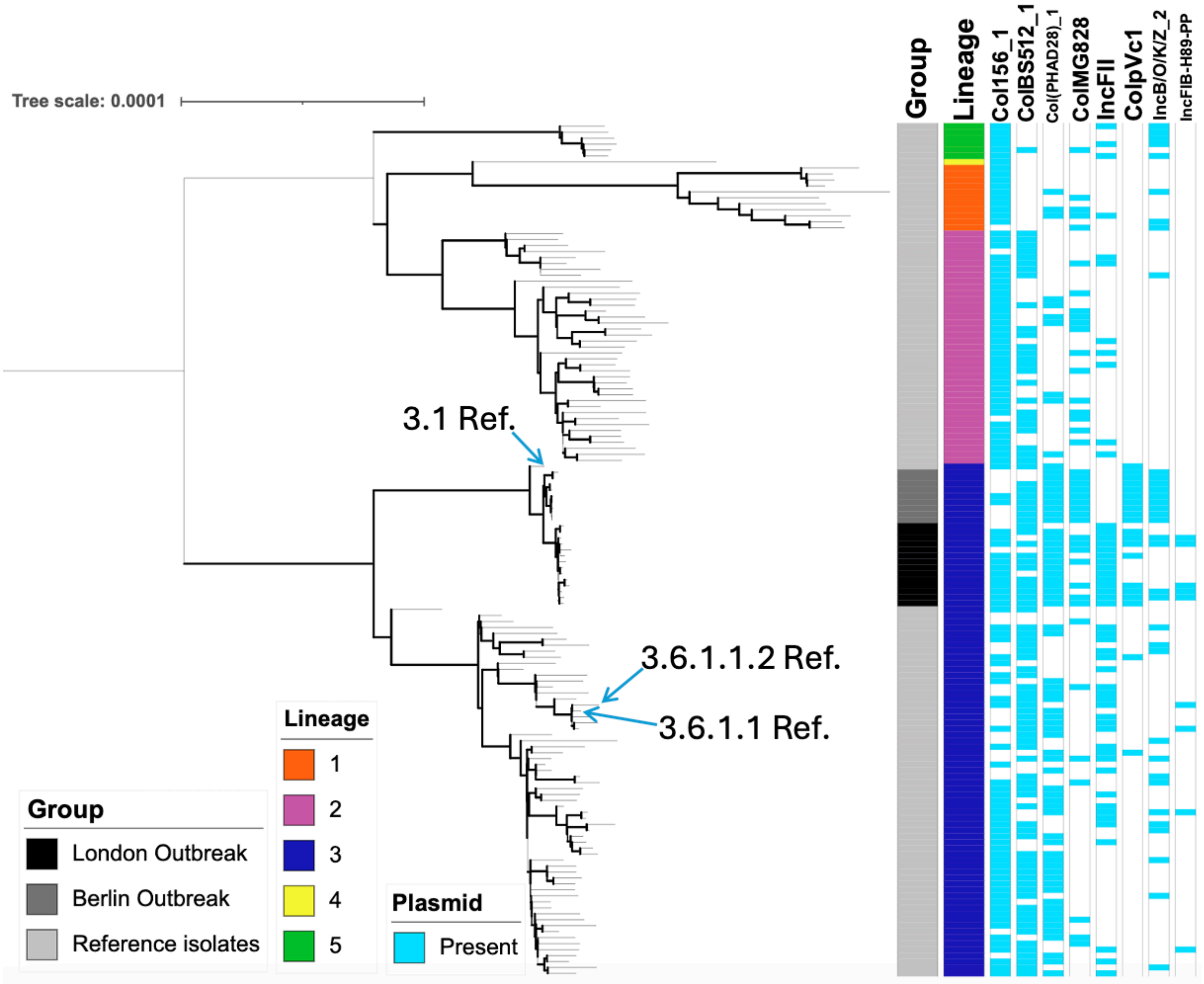

Supplementary Figure 4. Unique plasmid conservation profiles between the London and Berlin outbreak isolates. The tree from Figure 4 is shown with metadata tracks showing, group, lineage, and presence or absence of plasmids which change in proportion among groups of interest, coloured according to the inlaid keys.

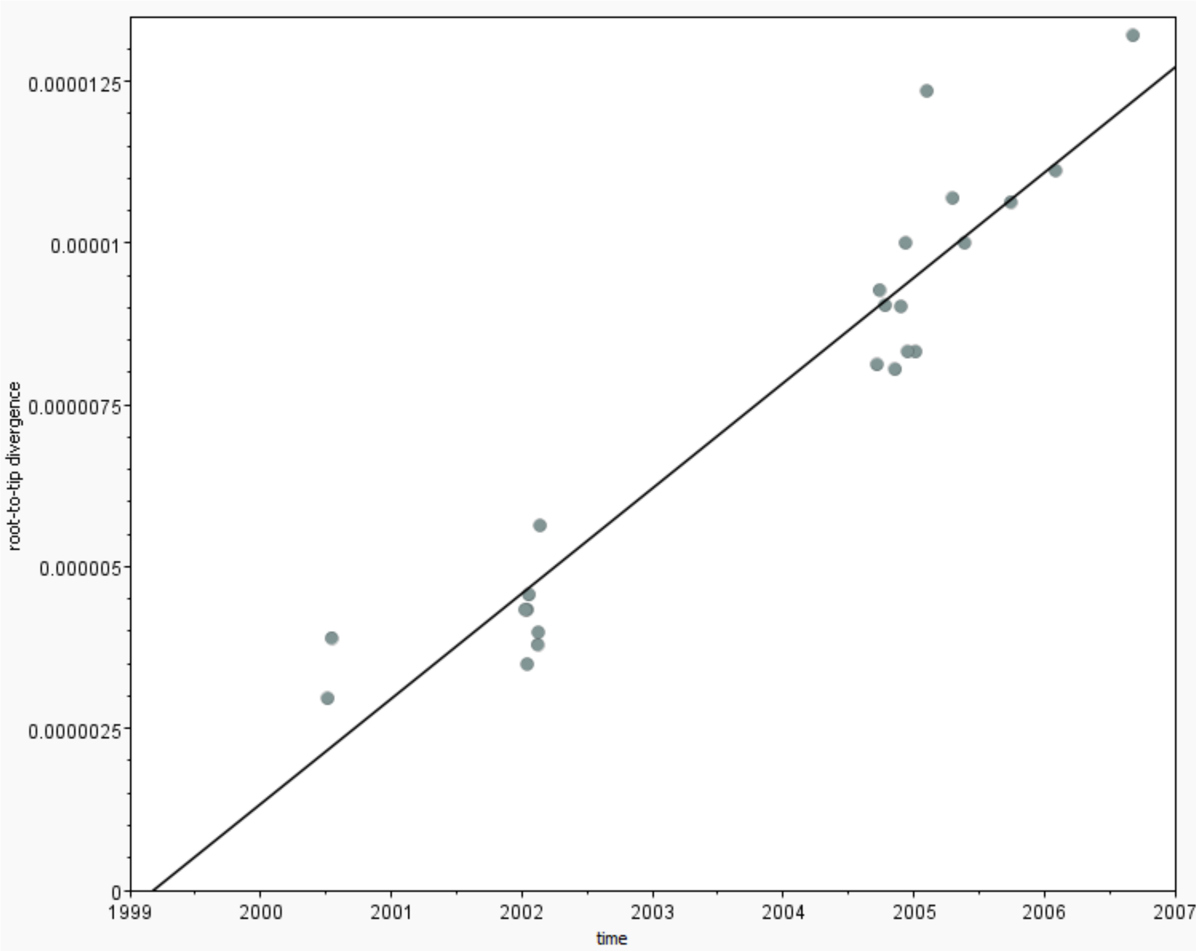

Supplementary Figure 5. The temporal signal of the Berlin and London outbreak isolates shows most recent common ancestor evolutionary emergence as the year 1999. Root-to-Tip divergence results showing the best-fitting root, heuristic residual mean squared. Input was a Newick tree file containing the isolates of interest. Slope (rate): 1.6231E-6; X-Intercept (TMPRCA) 1999.1688; Correlation Coefficient 0.9496; R squared 0.9017, residual mean squared 1.0246E-12. The specific date (dd/MM/yyyy) of collection for all the isolates were known. Dates were specified as years since 'sometime in the past'.

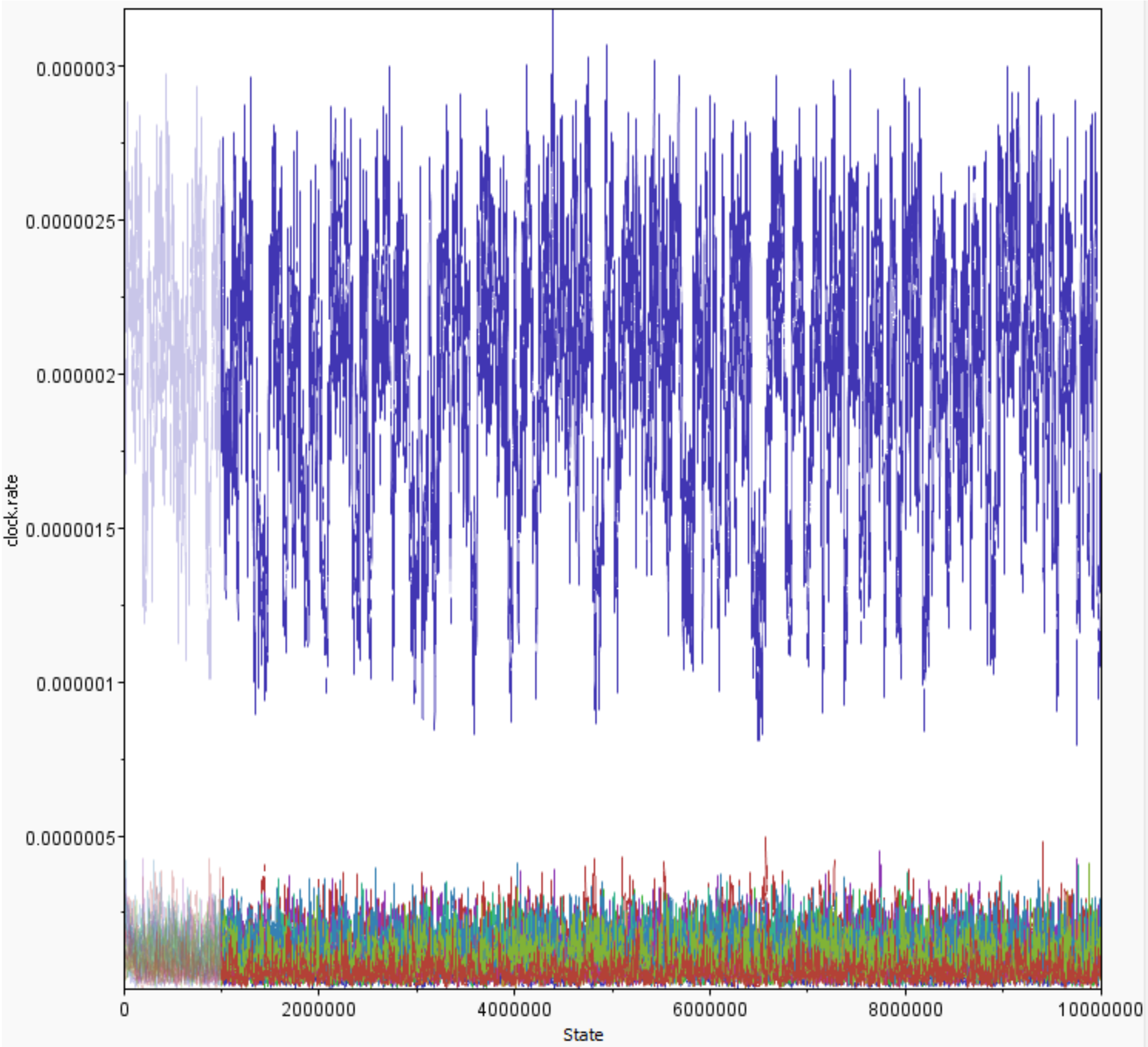

Supplementary Figure 6. The clock rate traces for the 'true' base run (dark blue trace above), compared to the ten randomised date runs (multicoloured traces below). The faded traces on the left are part of the 10% 'burn-in'.

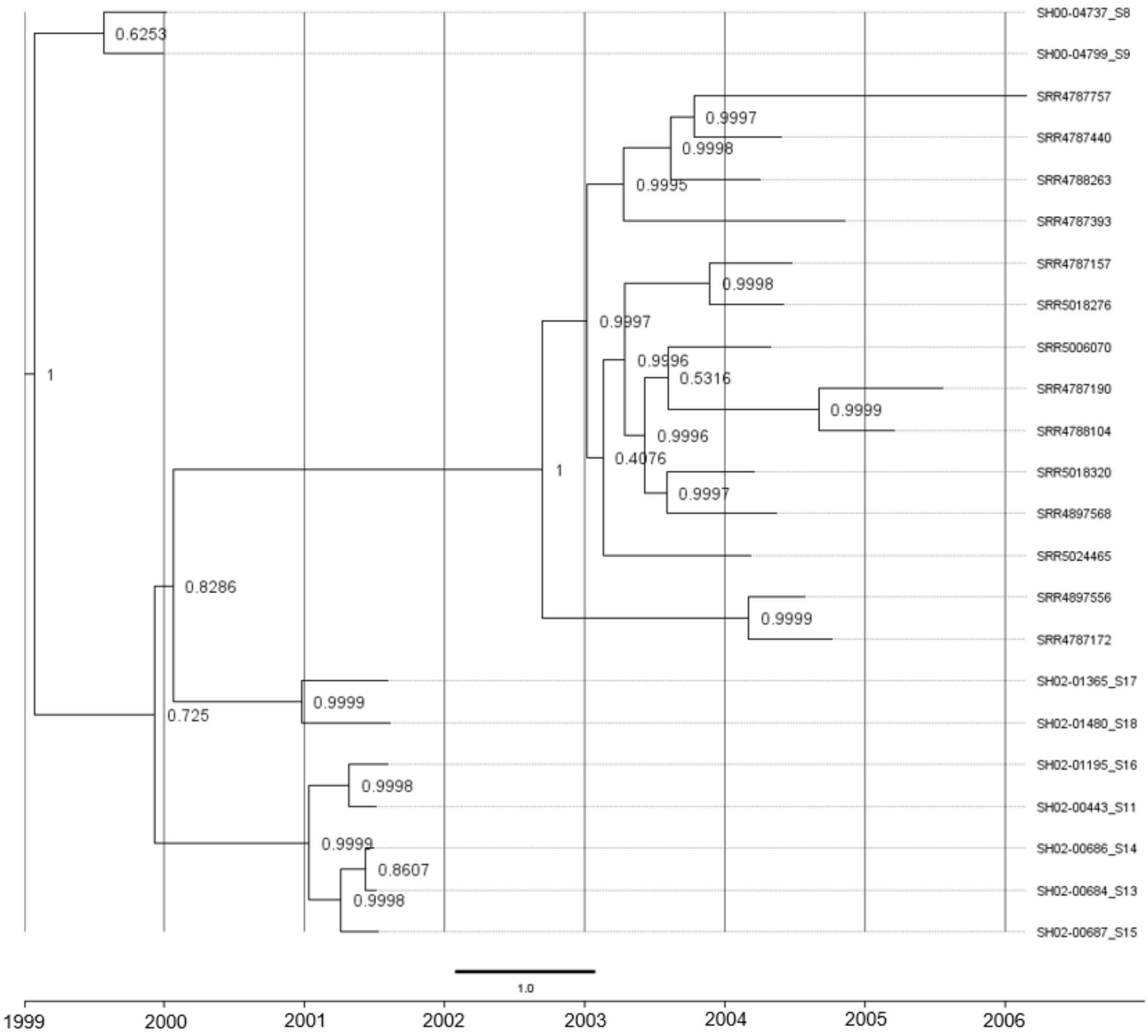

Supplementary Figure 7. The phylotemporal tree output from BEAST of the London and Berlin outbreak isolates. The scale bar is equal to exactly one year. Posterior is displayed on nodes. The x-axis labels have been manually converted from ‘years (*n*) preceding the most recent isolate in the tree’ to intuitively readable calendar years.

Supplementary Table 1. Various clock and prior combinations analysed through path sampling, alongside their marginal likelihood estimates and Bayes Factor calculations. Ranked from one to four, where ‘one’ is the best model with the highest marginal likelihood.

**Clock**	**Prior**	**Marginal likelihood (ML)**	**Δ in ML**	**Bayes Factor**	**Rank**
Strict	Exponential	-1524.9568151547294	0	0	1
Relaxed	Exponential	-1525.1262166008548	-0.169	0.339	2
Strict	Constant	-1527.3543430257323	-2.398	4.795	3
Relaxed	Constant	-1528.3147903300771	-3.358	6.716	4
